# Supplementary material for: Biochemical characterization of RecBCD enzyme from an Antarctic Pseudomonas species and identification of its cognate Chi (χ) sequence
Source: PLoS One. 2018 May 18;13(5):e0197476. doi: 10.1371/journal.pone.0197476 (PMC5959072; doi:10.1371/journal.pone.0197476)
Supplement: S1 File — (PDF) [file pone.0197476.s001.pdf]

S1 File
Supporting Figures and Tables

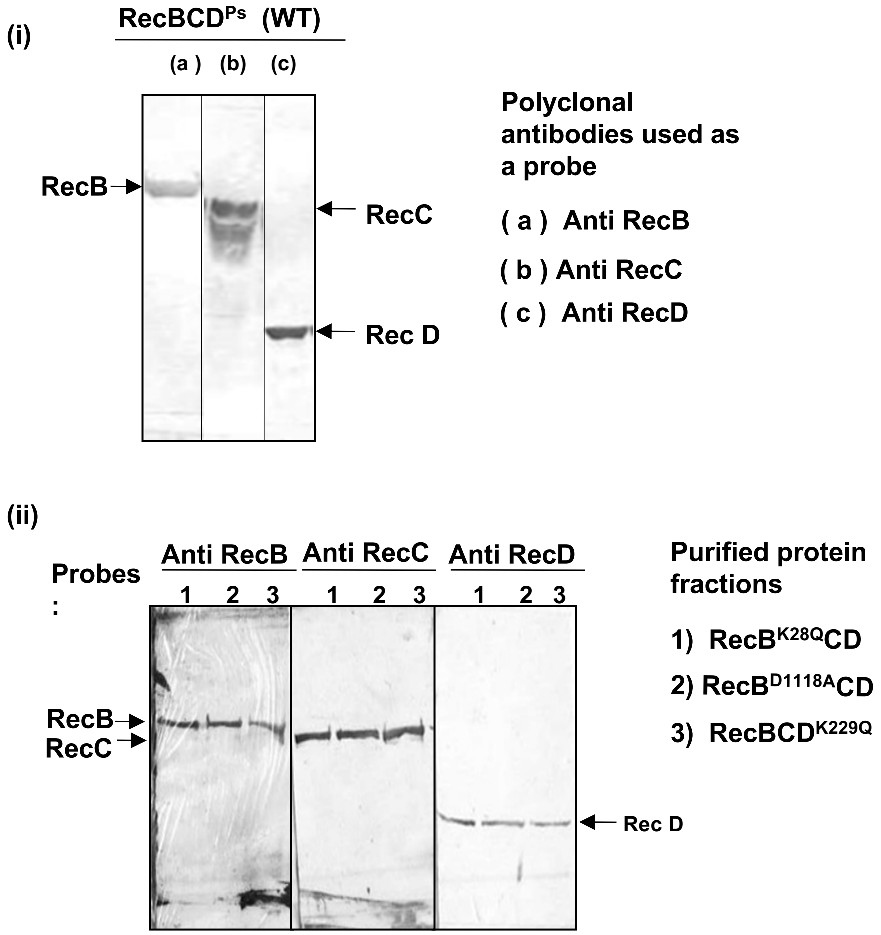

Fig A. Western analyses of protein wild-type and mutants RecBCD protein fractions. (i) Purified protein fractions of wild-type RecBCD enzyme were probed with (a) anti-RecC, (b) anti-RecB, and (c) anti-RecD antibodies as indicated. **(ii)** Purified protein factions of 1) $RecB^{K28Q}CD$, 2) $RecB^{D1118A}CD$, and, 3) $RecBCD^{K229Q}$ enzymes probed with anti-RecB, anti-RecC, and anti-RecD antibodies as indicated. The protein bands corresponding to RecC, RecB, and RecD are indicated by arrows.

ATP	-	-	0	2	2	2	2	2	2	2	2	2 mM
Mg^{++}	-	-	6	0	6	6	6	6	6	6	6	6 mM
Ca^{++}	-	-	0	6	0	2	4	6	8	12	18	24 mM
Lane	C_1	C_2	1	2	3	4	5	6	7	8	9	10

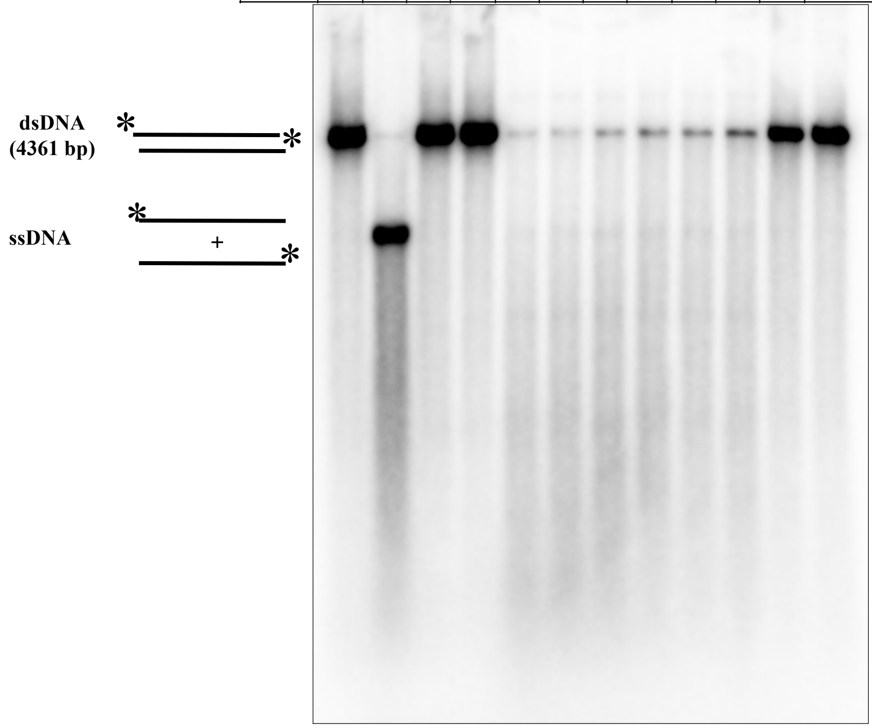

Fig B. Effect of Ca^{++} on unwinding and degradation of 5`-end labeled *Nde*I linearized pBR322 by RecBCD enzyme. The effect of Ca^{++} on unwinding and degradation of linearized pBR322 DNA was studied by varying the concentration of Ca^{++} in a reaction mixture that contained 25 mM Tris-acetate (pH 7.5), 2 mM ATP, 6 mM magnesium acetate, 1 mM DTT, 10 μM (nucleotides) linear pBR322 dsDNA (2.25 nM DNA end), 2 μM SSB protein and 0.5 nM RecBCD. The concentration of Ca^{++} in the reactions has been indicated. The lanes C_1 and C_2 contain 5'-^{32}P labeled linearized double-stranded and the heat-denatured linear pBR322 DNA.

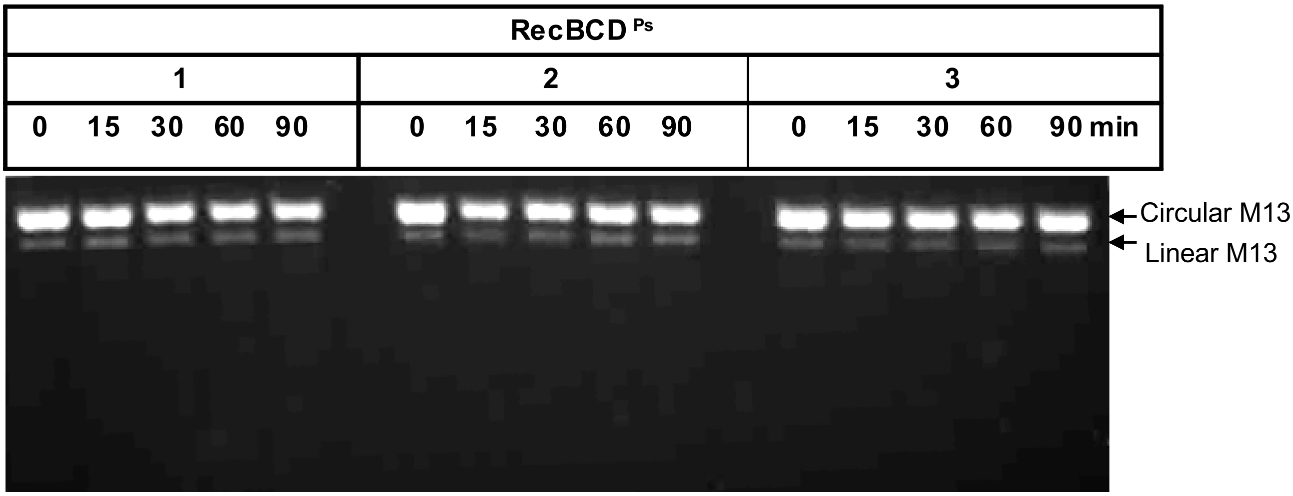

Fig C. Endonuclease activity of RecBCD^{Ps} on circular single-stranded DNA. The endonuclease activity of RecBCD enzyme was tested in 3 different buffer conditions: (1) The first reaction mixture contained 50 mM MOPS (pH 7.5), 10 mM MgCl2, 4.16 nM circular M13 ssDNA and 0.5 nM RecBCD; (2) Reaction mixture contained 25 mM Tris-acetate, 1 mM ATP, 8 mM Mg-acetate, 1 mM DTT, 4.16 nM M13 ssDNA and 0.5 nM RecBCD; (3) Reaction mixture contained 25 mM Tris-acetate, 2 mM ATP, 6 mM Mg-acetate, 1 mM DTT, 4.16 nM M13 ssDNA and 0.5 nM RecBCD. Samples were removed at the indicated times, and the reactions were quenched with 120 mM EDTA, 40% (v/v) glycerol, and 0.125% bromphenol blue, and analyzed on a 0.8% agarose gel in 1X TBE (90 mM Tris borate, 2 mM EDTA). The gel was run at 4 V/cm for 3 h and stained with ethidium bromide (0.5 mg/ml). The bands were visualized by exposure to UV light.

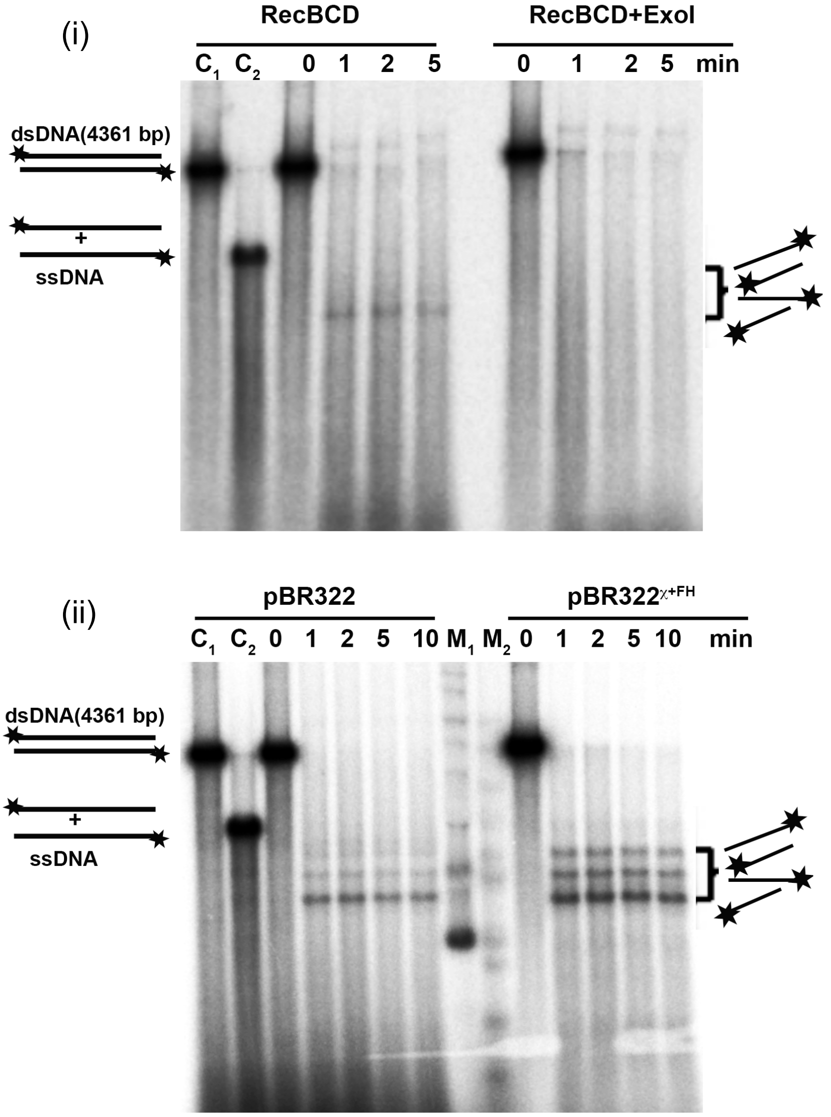

Fig D. (i) Effect of ExoI on the discrete DNA fragments produced by the nuclease activity of RecBCD^{Ps} enzyme. The nuclease reactions (25 mM Tris-acetate (pH 7.5), 2 mM ATP, 6 mM Mg acetate, 1 mM DTT, 10 μM (nucleotides) linear [5'-^{32}P] labeled pBR322 dsDNA and 2 μM SSB protein) were carried out in the presence of WT RecBCD^{Ps} enzyme (0.5 nM) (Left panel), or in the presence of both RecBCD^{Ps} and Exonuclease I (50 U) (right panel). The degraded DNA fragments of discrete size can be seen only in the reaction products containing wild-type RecBCD enzyme (Left panel), not in the reaction mixture containing both RecBCD and ExoI enzymes (Right panel).

(ii) DNA degradation activity of RecBCD^{Ps} on the *Nde*I digested linear dsDNA of pBR322 (*E. coli* χ^0) and pBR322$^{\chi+FH}$ (*E. coli* χ^+). Reaction mixture contained 25 mM Tris-acetate (pH 7.5), 2 mM ATP, 6 mM Mg acetate, 1 mM DTT, 2 μM SSB, 0.5 nM wild-type RecBCD enzyme, 10 μM (nucleotides) linear [5'-^{32}P] labeled pBR322 dsDNA (Left panel), and 10 μM (nucleotides) linear [5'-^{32}P] labeled pBR322$^{\chi+FH}$ dsDNA (Right panel) as substrates. The reactions were initiated by adding ATP and samples were removed at the indicated times, quenched by adding stop-buffer. The lanes C_1 and C_2 contain [5'-^{32}P] labeled *Nde*I linearized double-stranded and the heat-denatured ssDNA of pBR322, respectively. Notice that degraded ssDNA fragments produced from pBR322 and pBR322$^{\chi+FH}$ DNA by RecBCD^{Ps} were same. Lanes M1 and M2 contain double-stranded and single-stranded (heat-denatured) molecular size makers, [5'-^{32}P] labeled 1kb ladder (NEB), respectively.

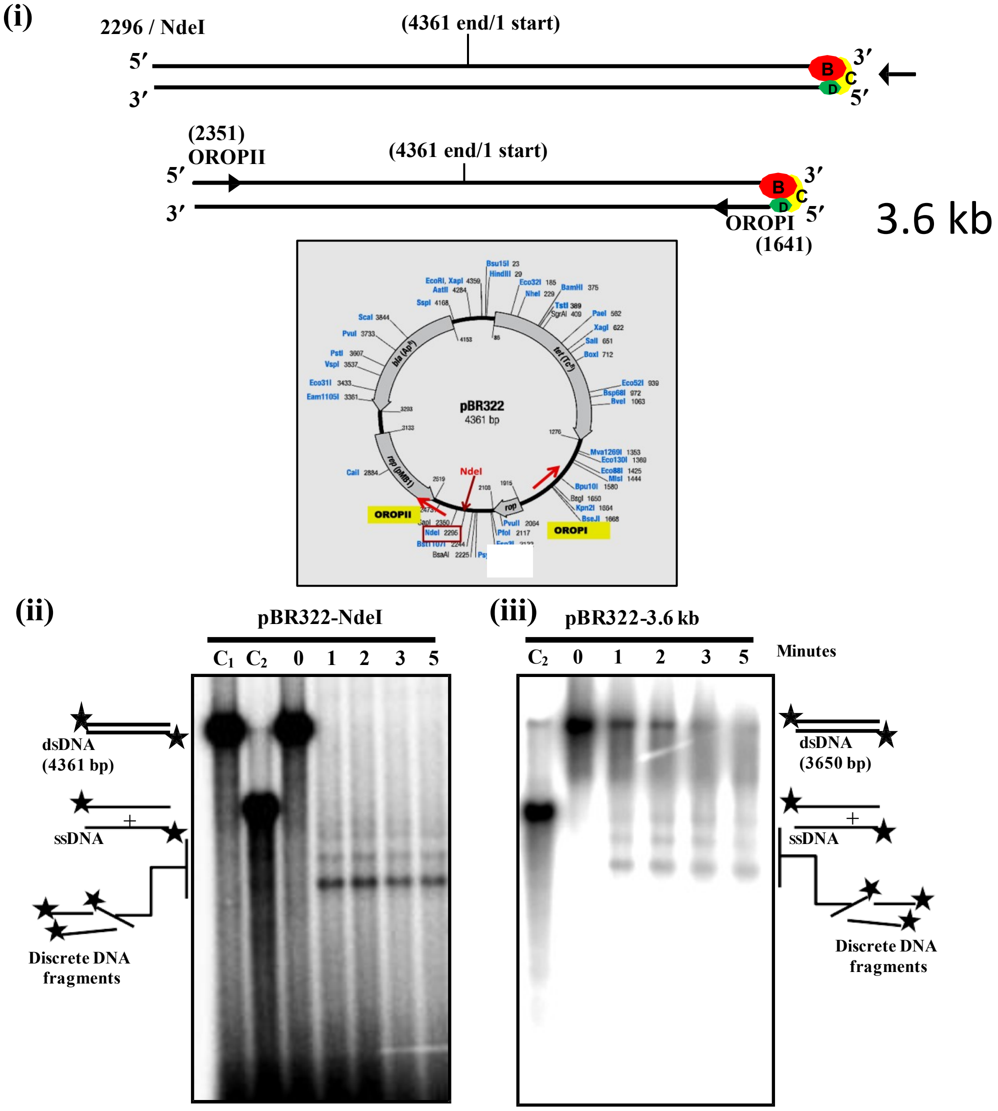

Fig E. DNA degradation activities of RecBCD^{Ps} on the linear dsDNA of pBR322. (i) Schematic of the location of primers used for PCR amplification and, the NdeI site which was used for preparation of linearized plasmid. Reaction mixture for assay contained 25 mM Tris-acetate (pH 7.5), 2 mM ATP, 6 mM Mg acetate, 1 mM DTT, 2 μM SSB, 0.5 nM wild-type RecBCD enzyme and 10 [illegible]M (nucleotides) in terms of DNA substrates. **(ii)** [5′-^{32}P] labelled NdeI digested pBR322 dsDNA (4.36 kb) or **(iii)** 10 μM (nucleotides) of 3.6 kb linear [5′-^{32}P] labeled PCR- amplified fragment (amplified by OROPI and OROPII primers) of pBR322, were used as substrates. The reactions were initiated by adding ATP and samples were removed at the indicated times, quenched by adding stop-buffer. The lanes marked with C_1 and C_2 contain the control samples for [5′-^{32}P] labeled double-stranded and the heat-denatured ssDNA of pBR322, respectively. Notice that three protected ssDNA fragments were observed with both pBR322 (4.36 kb) and 3.6 kb PCR amplified DNA by the RecBCD^{Ps} activity. The intensity of the lowest band was higher compared to the other two bands.

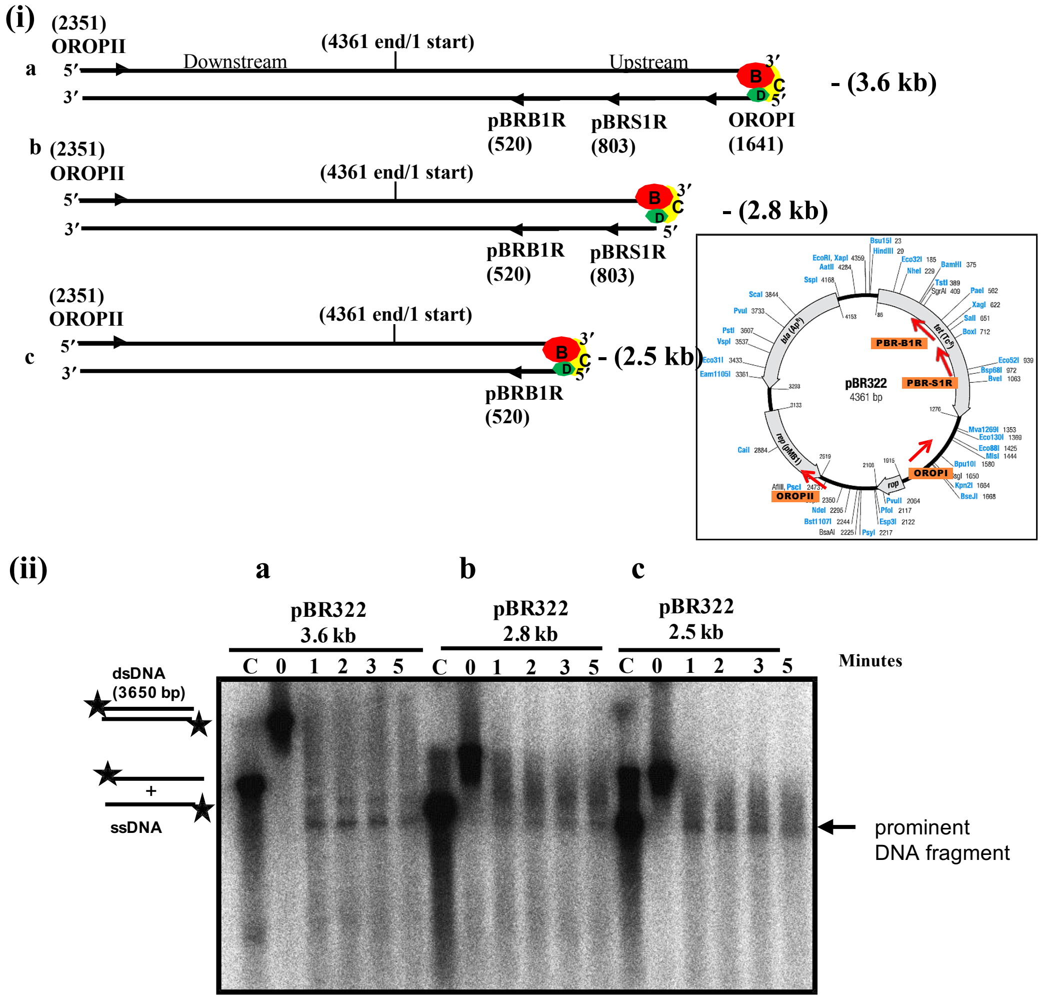

Fig F. Nuclease assays of RecBCD^{Ps} on the different amplified fragments of pBR322 to analyse the size of ssDNA fragments. Three fragments of different sizes were amplified from pBR322 using three sets of primers. **(i)** As shown above, OROPII and OROPI were used to amplify 3.6 kb (a), OROPII and pBRS1R to get 2.8 kb amplicon (b) and finally OROPII and pBRB1R were used to amplify 2.5 kb region of the plasmid (c). Inside the box, locations of primers are shown in pBR322 plasmid. **(ii)** RecBCD dependent DNA degradation assay was performed using PCR amplified DNA fragment of pBR322 as a substrate. The lane C contains [5'-^{32}P] labeled heat-denatured ssDNA of intact substrate DNA as a control for the respective experiments. DNA degradation activites of RecBCD^{Ps} enzyme on 3.6 kb DNA **(a)**, 2.8 kb DNA **(b)** and 2.5 kb DNA **(c)** as a substrate is shown here. Comparison of panel (a) and panel (c) clearly shows that the size of lowest intense ssDNA product (the prominent DNA band) is almost equal to the size of control DNA in the panel C (~2.5 kb). All numbers given in the brackets are the original nucleotide number of the plasmid pBR322 from where the primers have been designed. Total size of pBR322 plasmid is 4361 bp.

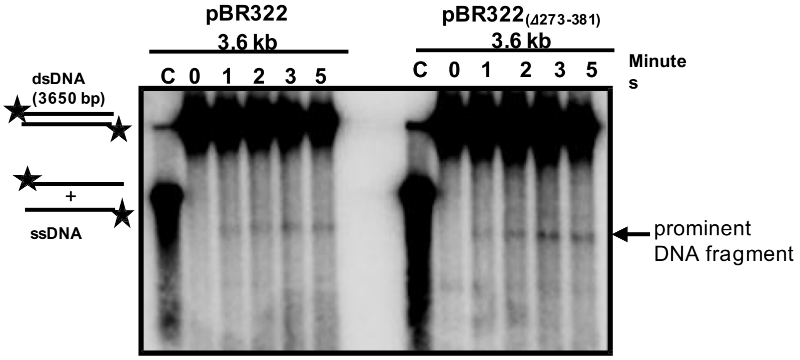

Fig G. Site directed deletion of pBR322 and analysis of ssDNA fragment products obtained from DNA degradation assay of RecBCD on these substrates. Assay was performed on pBR322 substrate having deletion of nucleotides from 273-381 nucleotide positions. It is to be noted that, though intense ssDNA product (the prominent DNA fragment) is still present while using pBR322 $_{(\Delta 273\text{-}381)}$ as a substrate but the size of this band is lower than the size of ssDNA produced using intact pBR322. This convinced us to speculate that putative Chi^{Ps} site will be in the upstream region of 381th nucleotide position.

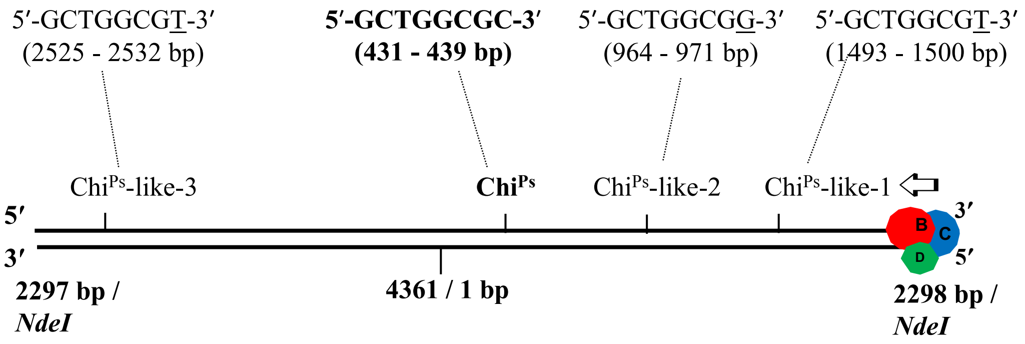

Fig H. Schematic representation of Chi^{Ps} and Chi^{Ps}-like sequences location on the NdeI linearized pBR322 plasmid. The RecBCD enzyme, when it enters from the 3′-side of NdeI-linearized pBR322 substrate (as indicated by the arrow), can potentially recognize Chi^{Ps} and Chi-like sequences. The Chi^{Ps} and Chi^{Ps}-like sequences and their corresponding position/s on pBR322 plasmid are indicated.

Table A

Plasmid derivatives used for studies related to Chi^{Ps} sequence identification		
pBR322	Plasmid with 'rep (pMB1)' replication origin, Amp^r, Tet^r	Fermentas life sciences
pBluescript SK (±)	Plasmid with pBR322 origin of replication, Amp^r	Stratagene (La jolla,CA)
$pBR322^{\Delta 273-381}$	pBR322 plasmid with deletion of nucleotide region from 273-381.	This study
$pBR322^{\Delta 400-450}$	pBR322 plasmid with deletion of nucleotide region from 400-450.	This study
$pBR322^{\Delta 401-419}$	pBR322 plasmid with deletion of nucleotide region from 401-419.	This study
$pBR322^{\Delta 421-439}$	pBR322 plasmid with deletion of nucleotide region from 421-439.	This study
$pBR322^{\Delta 421-429}$	pBR322 plasmid with deletion of nucleotide region from 421-429.	This study
$pBR322^{\Delta 431-439}$	pBR322 plasmid with deletion of nucleotide region from 431-439.	This study
$pBR322^{\Delta 441-449}$	pBR322 plasmid with deletion of nucleotide region from 441-449.	This study
$pBR322^{T971C}$	pBR322 plasmid containing a point mutation, which replaces the Thymine (T) nucleotide with Cytosine (C) at 971th position.	This study
$pBKS(Chi^{Ps})$	pBluescript plasmid containing an 8 mer sequence (5' GCTGGCGC 3') cloned at 2888th position.	This study

Table B

Primers used for site directed deletion in pBR322 and insertion of chi sequence in pBKS		
Primer	Sequence (5′-3′)	Related information
OROPI<br>OROPII	CTTCCGCGTTTCCAGACTTTACGAAACACG<br>CTCTTCCGCTTCCTCGCTCACTGACTCG	Primers to amplify 3.6 kb region of pBR322 for chi assay with RecBCD^{Ps} enzyme
DELPHIF<br>DELPHIR	ATTTCTATGCGCACCCGTTCTACGCCGGACGCATCGTG<br>CACGATGCGTCCGGCGTAGAACGGGTGCGCATAGAAAT	Primer set for site directed deletion of 273-381 region of pBR322
DELPHIF-2<br>DELPHIR-2	CTACGCCGGACGCATCGTGCATCACCGATGGGGAAGAT<br>ATCTTCCCCATCGGTGATGCACGATGCGTCCGGCGTAG	Primer set for site directed deletion of 400-450 region of pBR322
DELPHIF-3<br>DELPHIR-3	GCCGGCATCACCGGCGCCAATATCGCCGACATCACCGA<br>TCGGTGATGTCGGCGATATTGGCGCCGGTGATGCCGGC	Primer set for site directed deletion of 421-439 region of pBR322
DELPHIF-4<br>DELPHIR-4	CTACGCCGGACGCATCGTGAGGTGCGGTTGCTGGCGCC<br>GGCGCCAGCAACCGCACCTCACGATGCGTCCGGCGTAG	Primer set for site directed deletion of 401-419 region of pBR322
DELPHIF-5<br>DELPHIR-5	GCCGGCATCACCGGCGCCATGCTGGCGCCTATATCGCC<br>GGCGATATAGGCGCCAGCATGGCGCCGGTGATGCCGGC	Primer set for site directed deletion of 421-429 region of pBR322
DELPHIF-6<br>DELPHIR-6	CGGCGCCACAGGTGCGGTTATATCGCCGACATCACCGA<br>TCGGTGATGTCGGCGATATAACCGCACCTGTGGCGCCG	Primer set for site directed deletion of 431-439 region of pBR322
DELPHIF-7<br>DELPHIR-7	GGTGCGGTTGCTGGCGCCTACATCACCGATGGGGAAGA<br>TCTTCCCCATCGGTGATGTAGGCGCCAGCAACCGCACC	Primer set for site directed deletion of 441-449 region of pBR322
PBRphi2F<br>pBRphi2R	TACGTCTTGCTGGCGCTTCGCGACGCGAGG<br>CCTCGCGTCGCGAAGCGCCAGCAAGACGTA	Primer set for site directed mutation to change T to C at 971th position of pBR322
PBKSPHIF<br>PBKSPHIR	TTGTCTCATGAGCTGGCGCGGATACATATTTG<br>CAAATATGTATCCGCGCCAGCTCATGAGACAA	Primer set for cloning of Chi^{Ps} in pBluescript vector
